# High Erk-1 activation and Gadd45a expression as prognostic markers in high risk pediatric haemolymphoproliferative diseases

**DOI:** 10.1186/1756-9966-28-39

**Published:** 2009-03-19

**Authors:** Velia D'Angelo, Stefania Crisci, Fiorina Casale, Raffaele Addeo, Maria Giuliano, Elvira Pota, Paola Finsinger, Alfonso Baldi, Roberto Rondelli, Alberto Abbruzzese, Michele Caraglia, Paolo Indolfi

**Affiliations:** 1 Pediatric Oncology Service, Pediatric Department, F Fede, II University of Naples, Naples, Italy; 2 Department of Biochemistry and Biophysics, F Cedrangolo, II University of Naples, Naples, Italy; 3 Oncology Unit ASL Napoli 3, S Giovanni di Dio Hospital, Frattaminore, Italy; 4 Pediatric Onco-Haematology Department, Policlinico S Orsola-Malpighi, University of Bologna, Bologna, Italy

## Abstract

Studies on activated cell-signaling pathways responsible for neoplastic transformation are numerous in solid tumors and in adult leukemias. Despite of positive results in the evolution of pediatric hematopoietic neoplasias, there are some high-risk subtypes at worse prognosis. The aim of this study was to asses the expression and activation status of crucial proteins involved in cell-signaling pathways in order to identify molecular alterations responsible for the proliferation and/or escape from apoptosis of leukemic blasts. The quantitative and qualitative expression and activation of Erk-1, c-Jun, Caspase8, and Gadd45a was analyzed, by immunocytochemical (ICC) and western blotting methods, in bone marrow blasts of 72 patients affected by acute myeloid leukemia (AML), T-cell acute lymphoblastic leukemia (ALL) and stage IV non-Hodgkin Lymphoma (NHL). We found an upregulation of Erk-1, Caspase8, c-Jun, and Gadd45a proteins with a constitutive activation in 95.8%, 91.7%, 86.2%, 83.4% of analyzed specimens, respectively. It is worth noting that all AML patients showed an upregulation of all proteins studied and the high expression of GADD45a was associated to the lowest DFS median (p = 0.04). On univariate analysis, only Erk-1 phosphorylation status was found to be correlated with a significantly shorter 5-years DFS in all disease subgroups (p = 0.033) and the lowest DFS median in ALL/NHL subgroup (p = 0.04). Moreover, the simultaneous activation of multiple kinases, as we found for c-Jun and Erk-1 (r = 0.26; p = 0.025), might synergistically enhance survival and proliferation potential of leukemic cells. These results demonstrate an involvement of these proteins in survival of blast cells and, consequently, on relapse percentages of the different subgroups of patients.

## Introduction

Aberrations in regulation of a restricted number of key pathways that control cell proliferation and cell survival are mandatory for tumour growth and progression. Deregulated cell proliferation and suppressed apoptosis are both essential for cell transformation and sustained growth.

Hematological neoplasia are considered "special tumors" for their high sensitivity to the occurrence of spontaneous and pharmacological apoptosis. These cancers origin by tissues that use apoptosis for the regulation of their physiological mechanisms. These considerations explain the high sensitivity of these diseases to chemotherapy. However, high risk haematologic disease subtypes, that display a worse prognosis, also exist.

Acute lymphoblastic leukemia (ALL), acute myeloid leukemia (AML), and non-Hodgkin Lymphoma (NHL) are common cancer in children and teenagers [1]. Current treatment approaches are tailored according to the clinical characteristics of the host, genotype of the blasts, and early response to therapy [2]. Although these approaches have been successfully used in improving the outcome, several children with high risk acute leukemia and stage IV NHL still relapse. Cell drug resistance and cell-signaling pathways could be involved as important determinants of chemotherapy failure [3]. Programmed cell death, or apoptosis, has emerged as a common mechanism by which cells respond to cytotoxic drugs. However, the signaling mechanisms that mediate drug-induced apoptosis are still widely unknown.

Mitogen-activated protein kinase (MAPK) signaling cascades trigger stimulus-specific responses in cells: in fact, ERK is associated to proliferation and differentiation of hematopoietic cells while C-Jun N-terminal kinases (JNKs) are involved in stress-induced apoptosis and are associated to T cell activation [4]. A recent study showed that the JNK inhibition, in T-cell and Hepatocellular carcinoma cell lines, induces anti-tumor activity by growth arrest and CD95-mediated apoptosis through a transcription-independent mechanism [5]. Upregulation of the Ras/Raf/Mek/Erk pathways and phosphorylation of the downstream target are frequently observed in adult ALL and AML specimens and are associated to worse prognosis. In addition, it has been reported that Erk1 activation may represent an independent prognostic factor for achievement of complete remission in ALL and AML patients [6,7].

Another crucial cell mechanism involved in leukemogenesis is an alterate DNA repair and cell cycle arrest. Gadd45 is one of several growth arrest, apoptosis and DNA-damage-inducible genes. Interestingly, recent reports have suggested that GADD45a and b proteins also function in hematopoietic cell survival against genotoxic stress, in apparent contradiction to the role that GADD45 proteins family plays in apoptosis of epithelial and endothelial cells [8]. These data indicated that, conversely to the pro-apoptotic function of GADD45, in hematopoietic cells both *Gadd45a *and *Gadd45b *genes play a survival role.

Induction of *Gadd45 *genes at the onset of myeloid differentiation suggested that Gadd45a protein plays a role in hematopoiesis [9].

Altered expression and activity of different components of the apoptotic pathway, including receptors, ligands, adaptors, and caspases, can contribute to malfunction of the apoptotic machinery and, ultimately, to a more malignant phenotype. The ability of cytotoxic agents to trigger caspase activation appears to be a crucial determinant of drug response [10,11].

At our knowledge, the role of these molecular markers in prognosis determination of malignant pediatric haematologic neoplasms is still under-investigated. Moreover, novel treatment modalities have been directed towards inappropriately activated cell-signaling pathways that may be responsible for the proliferation and/or escape from apoptosis of leukemic blasts [12]. For this reason, the aim of the present study was to evaluate the expression and activity of cell-signaling-related proteins in blasts of children and teenagers affected by high risk haematologic neoplasms, such as AML, T cell ALL and stage IV NHL characterized by bone marrow infiltration. These molecular features have been subsequently correlated to the clinical outcome and to other biological prognostic factors.

## Materials and methods

### Patients

Seventy-two children with T cell ALL (18 samples), AML (45 samples) and stage IV NHL (9 samples) diagnosed and treated at the Oncology Pediatric Service of the Second University of Naples were enrolled in this study. The diagnosis was established by cytological examination of bone marrow smears and cytochemical tests included the staining for Periodic Acid Shiff (PAS), Myeloperoxidase (MPO), Alpha-Naphthyl-Acetate Esterase (ANAE) and Acidic Phosphatase (ACP). All samples presented a percentage of blast cells > 90%.

The patients with acute leukemias (AL) were sub-classified as ALL or AML according to the French American British (FAB) classification [[24], 25, 26] and NHL patients according to the NCI classification according to "Working Formulation". All NHL patients were stage IV for bone marrow involvement. The AML patients were treated according to AIEOP-AML protocols ('87, '92, '01–'02), ALL and NHL patients according to AIEOP-ALL protocols ('95, '00) [13].

### Immunocytochemistry

The bone marrow slides, collected at diagnosis, were fixed in acetone-methanol solution (1:1 dilution) for 30 seconds at 4°C. Mouse anti-human monoclonal antibodies raised against JNK phosphorylated on Serine-63, anti-Caspase8 p20 for p-20 subunit, anti-human Gadd45a (amino acids 1–165) and anti-pErk-1 phosphorylated on Tyrosine-204 were purchased from Santa Cruz Biotecnology (Santa Cruz, CA). All the primary antibodies were used at 1:100 dilution and added to the slides for 30 minutes at 37°C.

After three washes in Tris buffer, the Alkaline Phosphatase-conjugated Envision System DAKO was used to visualize the sites of localization of the different proteins expressed in bone marrow cells. This kit is unaffected by endogenous Alkaline Phosphatase activity because includes as blocking reagent levamisole and shows high sensitivity. Fast Red was used as the final chromogen. Cells were counterstained with Mayer's hematoxylin solution. HL60 cell-line cytocentrifuged slides were used as positive controls. Negative controls for each reaction were performed leaving out the primary antibody. Stained slides were analyzed for percentage of positive cells by two independent investigators. All samples were processed under the same conditions. The specificity of staining was also confirmed by competition of the primary antibodies with the respective peptide against which they were generated (data not shown).

The staining pattern of the four proteins was evaluated separately and the protein expression was scored in each specimen for the percentage of positive neoplastic cells: score 0 = undetectable staining; score 1 = from 1 to 30% of positive cells; score 2 = more than 30% of positive cells. Written informed consent was obtained from the parents.

Analysis of the data using such arbitrary cut-offs was statistically significant and, therefore, functionally operative.

The intensity of the staining was also evaluated for all proteins and scored in low and intermediate/high intensity compared with the 3+ Bcl-2 intensity of staining of the background lymphocytes to produce a semiquantitative evaluation of the immunostaining as previously described (Wang Y, Kristensen GB, Helland A, Nesland JM, Borresen-Dale AL, Holm R. 2005. Protein expression and prognostic value of genes in the erb-b signaling pathway in advanced ovarian carcinomas. Am J Clin Pathol 124:392–401.).

#### Western blot analysis

For cell extract preparation, the blasts were washed twice with ice-cold PBS/BSA, scraped, and centrifuged for 30 min at 4°C in 1 ml of lysis buffer (1% Triton, 0.5% sodium deoxycholate, 0.1 NaCl, 1 mM EDTA, pH 7.5, 10 mM Na_2_HPO_4_, pH 7.4, 10 mM PMSF, 25 mM benzamidin, 1 mM leupeptin, 0.025 units/ml aprotinin). Equal amounts of cell proteins were separated by SDS-PAGE. The proteins on the gels were electro-transferred to nitrocellulose and reacted with Rabbit antisera raised against α-tubulin, pErk-1/2 K-23, and Erk C-14 purchased from Santa Cruz Biotechnology (Santa Cruz, CA).

### Statistical analysis

Standard statistical description of parameters were used to characterize the data (mean, median and range). Spearman correlation test or chi-square test was used to assess the relationship between clinical parameters and immunocytochemical data. All p values are two-sided and values less than 0.05 were considered statistically significant.

Disease free survival (DSF) probability was calculated by Kaplan Meier method; comparison between probabilities in different groups was performed using the log-rank test. In DFS analysis, relapse and death due to any cause were considered treatment failures. DFS was calculated for all patients that obtained complete remission from the date of remission to relapse, death or date of last follow-up.

The remission status of the patients was determined on morphologic bases and complete remission was defined as less than 5% blasts in a normocellular bone marrow. Complete disappearance of all visible disease was required for NHL patients. If complete remission was not achieved (resistant patients) DFS was recorded as 0.

In the univariate analysis of DFS, the following variables were evaluated: gender, age, white blood cells at diagnosis and type of hematological neoplasia.

## Results

### Patient characteristics

Seventy-two patients (24 females and 48 males) were included in this study. The age of patients ranged from 0.5 to 13.7 years (median, 6.2 years). Clinical and pathological data of the 72 patients are listed in table 1. Eighteen patients (25%) had T cell ALL, forty-five (62.5%) had AML (no M3 subtype) and nine (12.5%) had stage IV NHL disease. At presentation, forty-one patients (57%) had white blood cells (WBC) higher than 20,000/mmc and thirty-one (43%) a lower count. Morphologically, the AML patients were classified as M0 (1 case), M1 (5 cases), M2 (18 cases), M4 (10 cases) (two of which were secondary leukemia), M5 (8 cases), M6 (1 case), M7 (2 cases); T-cell ALL cases as L1 (1 case) and L2 (17 cases). The NHL patients were classified as Burkitt-like (1 case), T-cells (3 cases) and B-cells (5 cases) (14) (tab. 1).

**Table 1:** Clinical characteristics of patient enrolled in the study

**Variable**	**No. of samples**	**%**
*AGE*		
≤ 24 months	10	13.9
> 24 months	62	86.1

*SEX*		
MALES	48	65.3
FEMALES	24	34.7

*WBC*		
< 20000/mmc	31	43
≥ 20000/mmc	41	57

Tumour type		
AML	45	
M0	1	1.4
M1	5	7
M2	18	25
M4	10	13.8
M5	8	11
M6	1	1.4
M7	2	2.8
		
ALL-Tcells	18	
L1	1	1.4
L2	17	23.6
		
NHL	9	
T cells	3	4.2
B cells	5	7
Burkitt	1	1.4

### Qualitative and quantitative analysis of Gadd45a, pErk-1, pJNK and Caspase 8

Table 2 summarizes the results of the immunocytochemical analysis related to % of blasts with protein activation and intensity of the staining.

**Table 2:** Distribution of protein activation or expression and staining intensity in blasts derived from haematological neoplasms

*Marker*	*Activated status* *Number of patients (%)*			*Staining Intensity* *Number of patients (%)*	
	negative	1–30%	>30%	Low	Intermediate/high
					
Gadd45a	12 (16.6%)	30 (41.7%)	30 (41.7%)	20 (33.3%)	40 (66.7%)
pErk-1	3 (4.2%)	22 (30.5%)	47 (65.3%)	13 (18.8%)	56 (81.2%)
JNK	10 (13.8%)	36 (50%)	26 (36.2%)	16 (25.8%)	46 (74.2%)
Caspase8	6 (8.3%)	32 (44.4%)	34 (47.3%)	21 (31.8%)	45 (68.2%)

**Figure 1:**
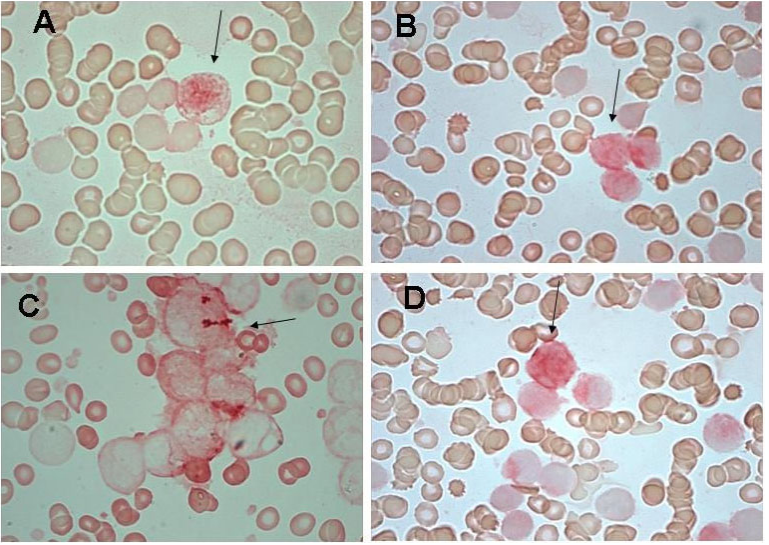
**Representative ICC for JNK (A), pErk-1 (B), Gadd45a (C) and Caspase8 (D)**. (A) JNK nuclear immune-reactivity in positive bone marrow blasts. (B, C) pErk-1 and Gadd45a nuclear and cytoplasmic staining in blasts. (D) Caspase8 cytoplasmic immune-staining in bone marrow blasts. Arrows show positive red stained cells.

In details, 30 specimens showed low and 30 high Gadd45a expression levels (83.4%), while in 12 samples (16.6%) the protein was absent. Immune-reactivity, detected in the nuclei and cytoplasms of blasts, showed high or low staining intensity in 40/60 samples (66.7%) and 20/60 samples (33.3%), respectively (Figure 1A).

Erk-1 activation, was detected in 69 of 72 evaluated specimens (95.8%): score 1 and 2 in 30.5% and 65.3%, respectively. The intensity of nuclear staining showed low or intermedie/high staining in 18.8% and 81.2% samples, respectively (Fig. 1B). JNK activation showed score 1 or score 2 in 50% (36/72) and 36.2% (26/72) samples, respectively. The p-JNK nuclear and cytoplasmic staining intensity was low or intermediate/high in 25.8% (16/62) and 74.2% (46/62) samples, respectively (Fig. 1C). Caspase8 was undetectable in 8.3% (6/72) and detected in 91.7% (66/72) samples, respectively. In details, score 1 or score 2 was detected in 44.4% (32 samples) and 47.3% (34 samples), respectively. Intermediate/high or low intensity cytoplasmatic staining of Caspase8 was detected in 68.2% (45/66) and 31.8% (21/66) samples, respectively (Fig. 1D and Table 2).

**Figure 2:**
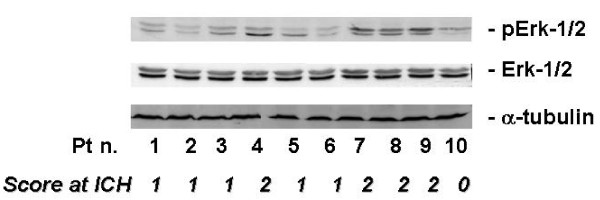
**Western blot assay for the expression of pErk 1 and 2 and total Erk 1 and 2**. The cells were processed for the determination of the phosphorylation and expression of Erk-1 and 2 evaluated after blotting with a specific anti-pMAPK and an anti-MAPK Mab, respectively, as described in "Materials and Methods". Expression of the house-keeping protein α-tubulin was used as loading control. In the same figure, the scores of the staining intensities of pErk-1 obtained at ICC in the same samples are also shown. The experiments were performed at least three different times and the results were always similar.

The statistical analysis of these data showed that the staining intensity of the four analyzed proteins directly correlated with the number of positive cells (p < 0.05). Moreover, we found a simultaneous activation of pJNK and Erk-1 in the evaluated blasts (r = 0.26; p = 0.025). In order to validate the results obtained in ICC, we have evaluated the expression of p-Erk-1 with western blotting with conventional antibodies used for the determination of p-Erk-1 and 2 and total Erk-1/2. The lower band shown in the gel, corresponding to a M.W. of 44 KDa, is clearly assessable in all the samples. The activity of the enzyme in the different evaluable patients strongly correlated to that one derived from experiments performed on blasts with ICC. An example of Erk-1 expression and activity on 10 different samples is now shown in Figure 2. Similar results were also obtained on all the other samples (data not shown).

### Protein activation levels in different groups subdivided for type of disease

When patients where subdivided into two different groups according to the diagnosis of neoplastic disease (ALL/NHL *vs *AML) we found a statistically significant difference of Gadd45a (p < 0.0001), pJNK (p = 0.0001), and Caspase8 (p = 0.004) between AML and ALL/NHL patients. Conversely, no difference in the phosphorylation of Erk-1 was detectable (p = 0.09). Interestingly, all AML patients showed an upregulation of the four studied proteins (Table 3).

**Table 3:** Proteins status in neoplasia subgroups

	**GADD45a**				**pERK-1**				**c-JUN**				**CASP ASE8**			
*Score*	0	1	2	p	0	1	2	p	0	1	2	p	0	1	2	p

ALL/NHL	12	12	3	**< 0.001**	3	10	14	0.09	10	10	7	**0.0001**	6	10	11	**0.004**

AML	0	18	27		0	12	33		0	26	19		0	22	23	

p values in bold are statistically significant.

### Correlation between constitutive proteins activation and outcome

At the time of this analysis 23 patients (31.9%) were alive in continuous complete remission, three patients (5.5%) alive in second complete remission, and 46 patients (63.8%) died from their disease.

Estimated 5-year DFS and OS rates for all patients were 31.9% and 36.1%, respectively.

By univariate analysis (log rank test) gender, age at diagnosis, WBC count and neoplasm type did not significantly influence the overall survival. The Kaplan-Meier method was used to assess any relationship between the studied molecular markers and patient survival time (Table 4). Erk-1 activation was confirmed to be an important prognostic factor (p = 0.033). However, the other molecular markers did not show any statistically significant correlation with overall survival.

**Table 4:** Outcome of patients studied and proteins status

	**GADD45a**				**pERK1**				**c-JUN**				**CASPASE 8**			
Score	0	1	2	*p*	0	1	2	*p*	0	1	2	*p*	0	1	2	*p*

**ALL/NHL**	12	12	3	**0.004**	3	10	14	0.8	10	10	7	0.9	6	10	11	0.8

Resistant/Relapse	12	0	0		3	6	9		10	8	7		6	7	9	

**AML**	0	18	27	0.9	0	12	33	0.5	0	26	19		0	22	23	

Resistant/Relapse	0	15	20		0	5	24		0	10	12	0.4	0	11	14	0.8

Score 0 = negative; 1 = 1–30% of positive cells; 2: > 30% of positive cells; p values in bold are statistically significant.

**Figure 3:**
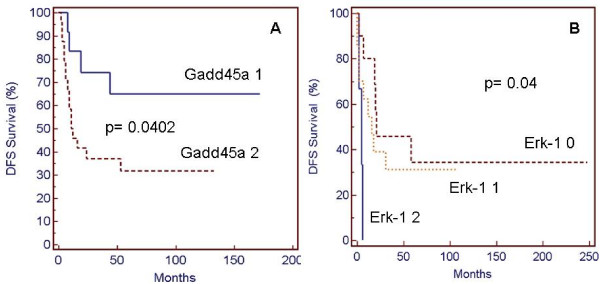
**Kaplan Meier DFS percentage plots in AML patients and ALL/NHL patients according to Gadd45a expression (A) and Erk1 activation level (B)**. Gadd45a 1: score 1; Gadd45a 2: score 2. Erk-1 0: score 0; Erk-1 1: score 1; Erk-1 2: score 2.

The analysis of DFS and of percentage of relapses in AML + NHL patients showed a statistically significant correlation with Gadd45a expression. In fact, patients with lower Gadd45a expression score had a better survival (p = 0.042) with a median DFS of 172 *vs *11,5 months for score 1 and 2, respectively (Fig. 3A). Similarly, the analysis of pERK1 score in the same group of patients revealed an inverse correlation between pErk-1 scores and DFS (p = 0.04). In fact, median DFS was of 5, 16 and 21 months in scores 3, 2 and 1, respectively (Fig. 3B).

## Discussion

The understanding of signals and pathways that regulate cell proliferation and apoptosis is crucial in searching for devices capable to treat cancer.

The knowledge of molecular bases of cancer has undoubtedly demonstrated that the inappropriate expression and activity of particular proteins involved in inter- and intra-cellular signaling networks causes radical changes in cell behaviour, enabling prolonged cell survival and unlimited proliferative capacity. [15,16] In this study we focused on the role of signal transduction pathways that control genomic stability and apoptosis in the prediction of clinical outcome of haematological malignancies. In details, the *in vivo *constitutive activation of Erk-1, JNK, Gadd45a and Caspase8 was evaluated in high-risk haemathological neoplasms. The immunocytochemical method was used for the investigation in order to avoid the contamination of the data by normal cells and allow the observation of protein status in single leukemic cells. It was found a constitutive activation of all the studied proteins especially in AML. Moreover, high Gadd45a expression (as both score and intensity) was associated with low DFS median. Our results suggest an alteration of the pathway that contributes to the maintenance of genomic stability by upregulation of Gadd45a [16]. To date, the involvement of Gadd45a in ALL has been observed only in vitro in leukemic cell lines [18]. In a previous study we observed that alteration of anti-apoptotic proteins such as Bcl-xl has been associated to increased tumour cell survival [23]. The present report shows, for the first time, that constitutive *in vivo *upregulation of Gadd45a in leukemic blasts promotes neoplastic hematopoietic cell survival that, based on our previous observations, probably occurs *via *p38 kinase and Bcl-xl. Another survival pathway over-activated in cancer cells is the Erk-1/2-mediated pathways and it was previously reported that Erk-1 activation may represent an independent prognostic factor for achievement of complete remission in ALL and AML patients [6,7]. We have indeed found that higher activation of this protein is a predictive marker of decreased overall survival in all diseases examined in the study and of reduced DFS in ALL/NHL subgroup. Interestingly, the staining intensity was correlated to the number of positive cells. This correlation clearly showed that an increase in the percentage of positive tumour cells correlates with a quantitative increase in protein phosphorylation in the leukemic elements. Activation of Erk-1 results in phosphorylation of many targets that have growth-promoting and pro-survival effects and it is not surprising that its activation correlates with a bad prognosis [19]. Moreover, in our series we observed an increased activation of JNK in 86% of patients (62/72) and the latter is involved in the stress-activated signaling cascades suggesting higher susceptibility of blasts to damage. Our results indicate that the activation of the signal transduction pathways components such as Erk-1 and JNK is very frequent in these poor prognosis subgroup disease. The simultaneous activation of multiple signaling pathways, might synergistically enhance survival and proliferation potential of leukemic cells protecting them from natural or pharmacologically-induced stress. In fact, the disruption of these signaling, is demonstrated to contribute to leukemogenesis by perturbing the rates of proliferation, differentiation and apoptosis [22-24]. In conclusion, this study confirms the relevant role of the MAPK pathway and/or other potentially involved signaling pathways in the pathogenesis and prognosis of high risk hematological diseases. Nevertheless, additional studies are required to define better the prognostic impact of these proteins. Moreover, small inhibitors targeting the MAPK pathway and/or other potentially involved signaling pathways, must be evaluated to establish whether this targeted therapeutic modality may add benefit (antiproliferative and/or pro-apoptotic) to current treatments of childhood leukemias and NHL, especially for high risk patients.

## Competing interests

The authors declare that they have no competing interests.

## Authors' contributions

VDA carried out the immunocytochemical studies and drafted the manuscript. SC carried out the western blots and collected the blasts. FC participated in the study design and clinical management of the patients. RA participated in the design of the study and preparation of databases. MG participated to the collection of the samples. EP participated to the collection of the samples and clinical data on follow-up. PF participated to immunocytochemical studies. AB interpreted and quantitized data derived from immunocytochemical studies. RR participated to the editing of the manuscript. AA managed the final stages of the study. MC participated to the drafting of the manuscript, the conclusion derivation and coordinated the western blotting studies. PI conceived of the study, and participated in its design and coordination. All authors read and approved the final manuscript.
